# Pin-Site Myiasis Caused by Screwworm Fly, Colombia

**DOI:** 10.3201/eid2105.141680

**Published:** 2015-05

**Authors:** Francisco J. Africano, Álvaro A. Faccini-Martínez, Carlos E. Pérez, Alejandro Espinal, Juan S. Bravo, Carlos Morales

**Affiliations:** Universidad Militar Nueva Granada, Bogota, Colombia (F.J. Africano); Servicios y Asesorías en Infectología, Bogota (Á.A. Faccini-Martínez);; Hospital Militar Central, Bogota (C.E. Pérez, A. Espinal, J.S. Bravo, C. Morales)

**Keywords:** myiasis, pin-site myiasis, Cochliomyia hominivorax, screwworm fly, larvae, parasites, femur fracture, external fixators, infection, infestation, surgery, Colombia

**To the Editor:** Myiasis is the infestation of humans or animals with dipterous insect larvae (*1*). The term pin-site myiasis was recently adopted for a rare and emerging parasitic infection after treatment of open fractures with external metal fixators (pins). Myiasis can also occur as a result of invasion of larvae deposited by flies in wounds adjacent to these fixators (*1*,*2*). We describe a patient with pin-site myiasis caused by the *Cochliomyia hominivorax* screwworm fly associated with external fixators used for treatment of an open fracture of the femur.

In September 2014, a 26-year-old male soldier from the Department of Meta in central Colombia was admitted to a primary medical unit for treatment of an open fracture of the right femur after a traffic accident. The patient had no relevant medical history. After multiple surgical interventions and external fixation of the fracture, he was discharged. Two weeks later, he returned to the medical unit with edema, redness, and warmth in the area surrounding the metallic fixators. At this time, 50 larvae were observed in the surgical wound (Figure, panel A).

**Figure:**
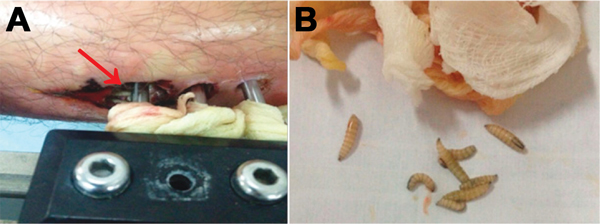
Pin-site myiasis in a 26-year-old male soldier, Colombia. A) Larvae of *Cochliomyia hominivorax* screwworm fly around an external metallic fixator (arrow). B) Larvae isolated from the insertion wound of the external metallic fixator

The patient was referred to Hospital Militar Central in Bogota, Colombia, where surgical cleansing of the wound was performed and 30 additional larvae were obtained (Figure, panel B). Extracted larvae were sent to the Parasitology Laboratory of the Universidad Nacional de Colombia in Bogota, Colombia for identification. The larvae were taxonomically classified as those of the *C. hominivorax* screwworm fly.

Treatment with oral ivermectin and intravenous ampicillin/sulbactam was initiated. The next day, surgical cleansing showed signs of osteomyelitis. A culture of bone tissue was positive for multidrug-susceptible *Pseudomonas aeruginosa* and *Stenotrophomonas maltophilia* susceptible to trimethoprim/sulfamethoxazole (TMP/SMX). At this time, antimicrobial drug therapy was changed to intravenous ciprofloxacin (400 mg every 12 h) and oral TMP/SMX (160/800 mg every 12 h). The patient completed 2 weeks of treatment in the hospital and showed no signs or symptoms of infection or infestation by larvae. He was discharged, prescribed oral TMP/SMX, and followed up by the Orthopedics and Infectious Diseases Service of Hospital Militar Central.

Bacterial infection in insertion sites of metallic pins is usually the most frequent complication when external fixators are used in treatment open fractures and represents 10%–40% of complications, followed by loss of pins (5%), pain/edema (3.3%), and vascular or nervous injury (1.7%) (*3*). In the past decade, pin-site myiasis has been described as a new complication; 6 cases have been reported (1 in the United States, 2 in Venezuela, and 3 in Greece) (*2**,**4**–**6*). All case-patients had predisposing risk factors for parasitic infestation, such as diabetes mellitus, immobilization, alcohol and drug use, or decreased immune status. Our patient had the same risk factor (previous surgical interventions) as that reported for a patient in Venezuela (*2*). Also, the anatomic region (leg) involved and the larvae species (*C. hominivorax*) identified for our patient were observed in other reported cases (*2**,**5**,**6*).

*C. hominivorax* screwworm fly is the main species involved in wound myiasis in the New World (*1*). Wound myiasis is initiated when female flies oviposit on or near a wound (<300 larvae/wound). Upon hatching, larvae, which have small spines on each body segment that resemble the threads of a screw, penetrate head first into the tissues, burrow deeper perpendicular to the skin surface (resembling a screw), and cause extensive destruction of tissue and a bloody discharge (*1*). *C. hominivorax* larvae differ from larvae of other fly species because they feed only on living flesh (*7*). The anatomic site around a lesion becomes swollen, and local tissue destruction can cause pain and secondary bacterial infection (*1*).

Our patient was co-infected with *P. aeruginosa*, which was similar to a patient with pin-site myiasis reported by Paris et al. (*5*). Removal of the metallic fixators (a necessary procedure in 50% of reported cases) (*1*) was not required for our patient. Surgical cleansing, extraction of all larvae, and antimicrobial drug therapy resulted in resolution of the infection.

After a screwworm eradication program was developed by the Animal and Plant Health Inspection Service of the US Department of Agriculture, screwworm was eradicated in the United States in 1966, in Mexico in 1991, in Belize and Guatemala in 1994, in El Salvador in 1995, in Honduras in 1996, in Nicaragua in 1999, in Costa Rica in 2000, and in Panama in 2006 (*7*). Current distribution of *C. hominivorax* screwworm flies is limited to South America and some Caribbean Islands (*1*). However, physicians should be aware of the possible reemergence of myiasis as a complication of surgery and use of metal fixators.
